# Supplementary material for: Tead1a Initiates Transcriptional Priming Through the TEAD1a/YAP‐Notch1‐Spi1/Cebpα Axis to Promote Neutrophil Fate
Source: Adv Sci (Weinh). 2025 Aug 28;12(41):e05441. doi: 10.1002/advs.202505441 (PMC12591127; doi:10.1002/advs.202505441)
Supplement: Supplementary file 4 — Supplementary Dataset [file ADVS-12-e05441-s004.zip › 3-1.zip!/3-1/02-1.cluster/cluster0/Enrich/ego_CC.pdf]

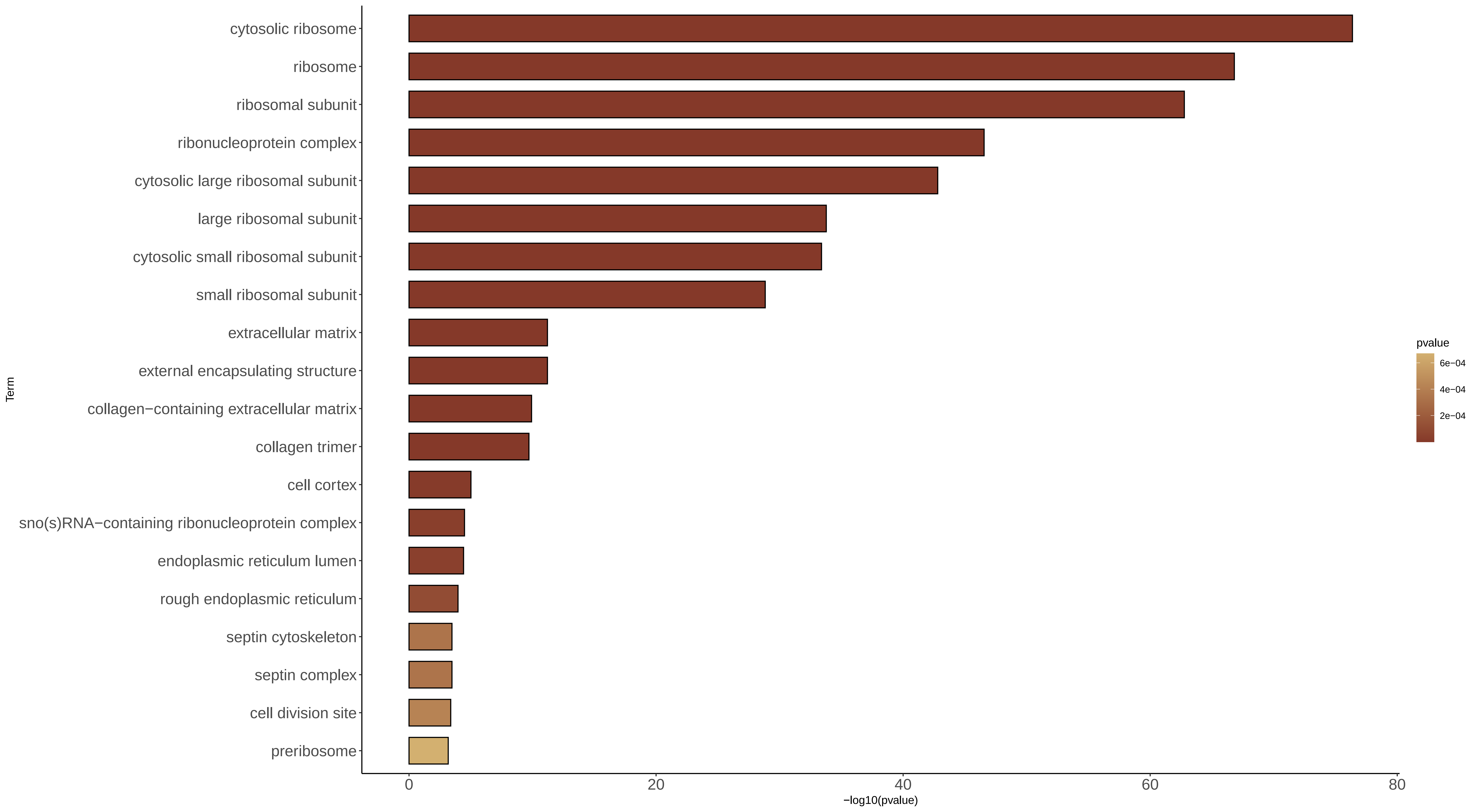
cytosolic ribosome
ribosome
ribosomal subunit
ribonucleoprotein complex
cytosolic large ribosomal subunit
large ribosomal subunit
cytosolic small ribosomal subunit
small ribosomal subunit
extracellular matrix
external encapsulating structure
collagen−containing extracellular matrix
collagen trimer
cell cortex
sno(s)RNA−containing ribonucleoprotein complex
endoplasmic reticulum lumen
rough endoplasmic reticulum
septin cytoskeleton
septin complex
cell division site
preribosome
Term
0
20
40
60
80
−log10(pvalue)
pvalue
6e−04
4e−04
2e−04
